# Utilization Potential of Aerated Concrete Block Powder and Coffee Grounds Ash in Green-Growing Concrete

**DOI:** 10.3390/ma17123027

**Published:** 2024-06-20

**Authors:** Jinping Li, Rong Huang, Zheng Chen, Xuedi Sun, Deliang Yu

**Affiliations:** 1 College of Materials Science and Engineering, Xi’an University of Architecture and Technology, Xi’an 710100, China; jinping@xauat.edu.cn (J.L.); sunxuedi@xauat.edu.cn (X.S.); ydl@xauat.edu.cn (D.Y.); 2 Shaanxi Construction Engineering Fifth Construction Group Co., Ltd., Xi’an 710100, China; huangrong@snwj.com

**Keywords:** aerated concrete block powder, coffee grounds ash, green-growing concrete, freezing resistance, porosity

## Abstract

The purpose of this research is to investigate the utilization potential of recycled powder made from spent coffee grounds (SCGs) and aerated concrete blocks (ACBs) in green-growing concrete. The green-growing concrete is prepared using ACB powder and SCG ash as raw materials instead of 5%, 15%, and 25% and 5%, 10%, and 15% cement, respectively. Then, the two raw materials are compounded with the optimal content. The compressive strength and alkalinity of green-growing concrete at 7d and 28d and the frost resistance after 25 freeze–thaw cycles at 28d are studied. The results showed that the optimum content of ACB powder and SCG ash was 5%. Replacing 5% cement with recycled powder could improve the strength of concrete. The alkalinity of concrete containing ACB powder gradually increased, while the alkalinity of concrete containing SCG ash gradually decreased. The alkalinity of ACB-SCG powder was lower than that of ACB powder but slightly higher than that of SCG ash. The frost resistance of concrete containing ACB powder decreased gradually, and the frost resistance of concrete containing SCG ash increased first and then decreased greatly. The frost resistance of ACB-SCG powder could neutralize that of ACB powder and SCG ash.

## 1. Introduction

With the rapid development of society and the continuous advancement of urbanization, the development of the construction industry has become increasingly fierce. At the same time, the treatment of construction waste (CWD) cannot be underestimated. The global annual output has exceeded 8 billion tons [1,2], which has caused great negative impacts on the environment. The effective treatment of CWD is crushing and screening it as recycled aggregate for concrete and mortar [3,4,5,6,7], which makes the use of construction waste widely welcomed. However, aerated concrete blocks (ACBs) and spent coffee grounds (SCGs) are challenging to recycle due to their high porosity [8,9,10]. Therefore, it has become a promising research direction for regenerating solid wastes such as ACBs and SCGs by grinding them into powder to remove pores as recycled powder.

ACBs are a new type of lightweight, porous building material widely used in buildings. Due to the advantages of being high-strength, having good thermal insulation, and being lightweight, they are generally used for infilled walls. The resulting waste ACBs are also increasing, and the proportion of waste could reach about 10% or even 15% [11]. Liu et al. [12] used waste aerated concrete block powder to replace 10%, 20%, and 30% of cement for experimental research and found that replacing 10% of cement with recycled powder could improve the strength of mortar. Qin [13] prepared PC-WAAC (Portland cement-waste aerated concrete) mortar specimens using the carbonization curing method [14]. It was found that the compressive strength of PC-WAAC specimens after carbonization curing was higher than that of PC specimens, and the optimum content of WAAC was 20%. Nasr et al. [15] found that adding 10% CCP (autoclaved aerated concrete block waste powder) could produce sustainable mortar, and the compressive strength was improved without a significant impact on other properties of the mortar. They used waste aerated concrete block powder to replace cement (<0.075 mm) or sand (0.075–0.15 mm) in mortar. Topcu [16] used crushed aerated concrete blocks instead of crushed stone as aggregates and discovered that it was appropriate to use waste autoclaved aerated concrete aggregates to produce lighter concrete than crushed stone concrete. However, there is no research on the preparation of green-growing concrete using aerated concrete block powder instead of partial cement.

In addition to ACBs, SCGs are also a major source of waste. Lin et al. [17] used coffee grounds ash at 500 °C and 600 °C instead of different amounts of cement to prepare mortar blocks, and found that the effect of replacing 10% of coffee grounds ash at 600 °C was better than other applications. Such utilization could also provide valuable economic and carbon dioxide emission reduction benefits. Choi et al. [18] replaced part of the cement in the cement mortar with coffee residue ash calcined at 800 °C and designed the mix ratio with the volume and gravimetric method, respectively. It was found that the coffee residue ash calcined at 800 °C (SCG_Ash) showed a hydration reaction, and the compressive strength was equivalent to or higher than that of OPC mortar. Wu et al. [19] used coffee grounds instead of partial cement to prepare plant-growing concrete and to study the mechanical and plant-growing properties. It was found that coffee grounds could effectively reduce the alkalinity of plant-growing concrete. When the amount of cement was 300 kg/m^3^, the water–cement ratio was 0.4, and the number of coffee grounds was 9 kg/m^3^, the performance of coffee grounds planting macroporous concrete was the best. Therefore, it seems to have great potential to use coffee grounds ash at 800 °C instead of partial cement to prepare green-growing concrete.

Green-growing concrete is an extraordinary new type of ecological concrete with a particular environmental effect or specific ecological function [20]. It takes porous concrete as the skeleton, fills the pores of porous concrete with suitable materials, plants seeds for planting, and covers the surface of the concrete so that plants and concrete are integrated [21]. Compared with traditional concrete, it has the functions of dust removal and noise reduction, water and air permeability, water purification, and heat storage, and has environmental friendliness or biocompatibility [22,23]. As an environmentally friendly material with certain strength and vegetation coverage, it is widely used in slope protection, riverbank slope protection, parking lot ground, and three-dimensional greening [23]. Kong et al. [24] used waste oyster shells (WOSs) and recycled aggregate (RA) to prepare artificial reefs with porous ecological concrete (PEC). It was discovered that WOS content should not go above 20%. RA can fully replace the natural aggregate (NA) in PEC to suit the needs of creating artificial reefs. Zhao et al. [25] employed magnesium ammonium phosphate cement (MPC) instead of OPC to manufacture a new type of porous ecological concrete. It was discovered that as the phosphorus/magnesium molar ratio (P/M) grew, the pH value of MPC progressively increased as well. The compressive strength reached its maximum of 49.2 MPa when the P/M value was 1/4. Wang et al. [26] stated that when the volume ratio of sandstone to limestone was less than 0.32:0.68 (i.e., the replacement rate of sandstone to limestone in ecologically permeable concrete was 32%), the best permeability coefficient was 9 mm/s. The strength of environmentally porous concrete satisfies 5 MPa requirements. However, there is no research on the use of coffee grounds as recycled powder instead of traditional building materials to improve the performance of green-growing concrete.

In this paper, on the basis of previous studies, through the treatment of solid waste ACBs and SCGs, the special properties of ACBs and SCGs are used to replace part of cement to save the cost of concrete and reduce the environmental pollution caused by CO_2_ emissions. ACBs and SCGs were used as recycled powder instead of partial cement to prepare the green-growing concrete to improve its performance. The alkalinity in the pores of green-growing concrete is a key factor affecting plant growth. The higher the alkalinity is, the less conducive to plant growth. Therefore, it is necessary to control the alkalinity in the pores of planting concrete. The study examined the impact of varying concentrations of ACB and SCG recycled powder, as well as their combined addition of 5%, on the alkalinity and compressive strength of green-growing concrete at 7d and 28d, the porosity following a 28d curing period, and the resistance to frost following 25 cycles of freeze–thaw, in order to improve the performance as much as possible under the premise of ensuring its strength and planting. The SEM images of ACB powder concrete, SCG ash concrete, and ACB-SCG powder concrete were analyzed via scanning electron microscopy. It provides a reference for the recycling of waste and the preparation of high-performance concrete.

## 2. Materials and Methods

### 2.1. Raw Materials

**Figure 1:**
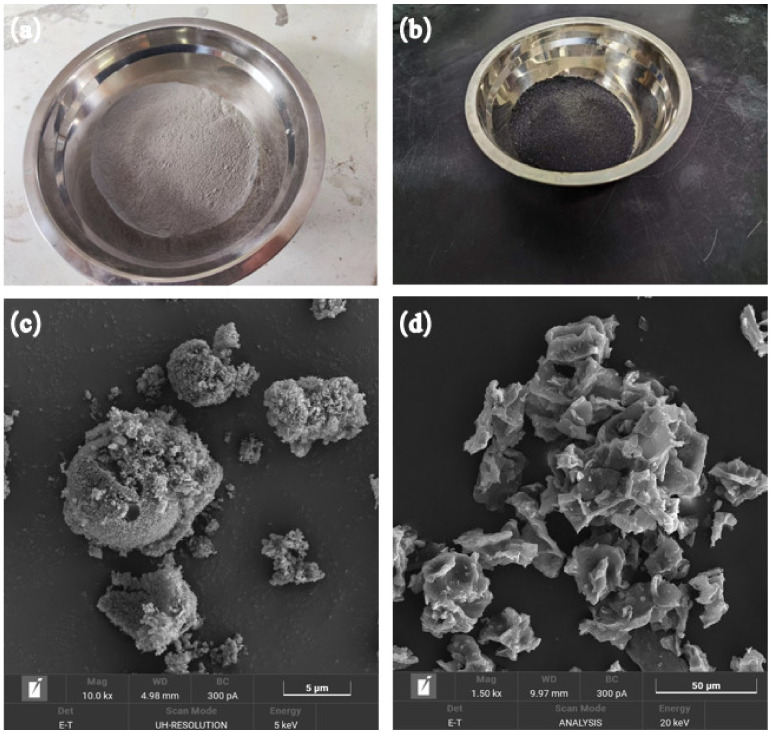
ACB powder and SCG ash diagram and SEM diagram: (**a**) ACB powder; (**b**) SCG ash; (**c**) the SEM of ACB powder; and (**d**) the SEM of SCG ash.

**Figure 2:**
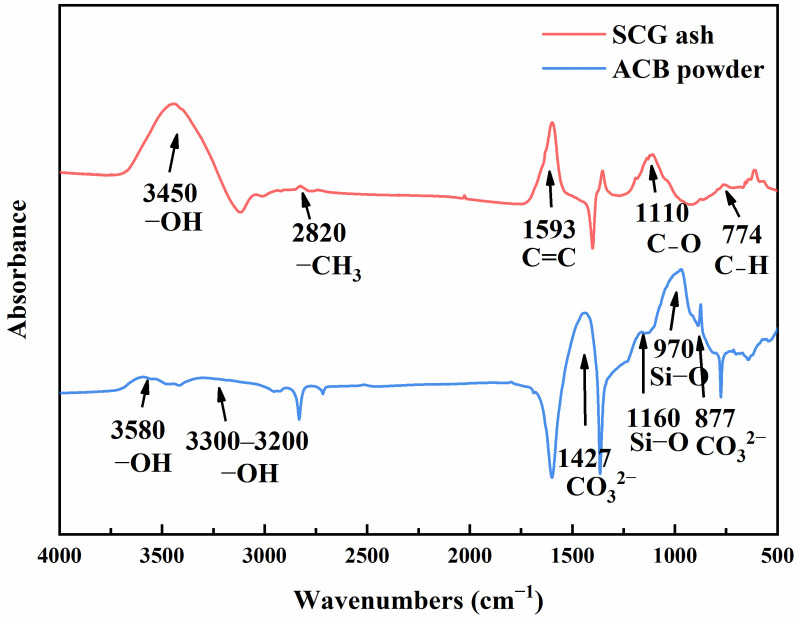
The FTIR test of recycled fine powder concrete.

**Figure 3:**
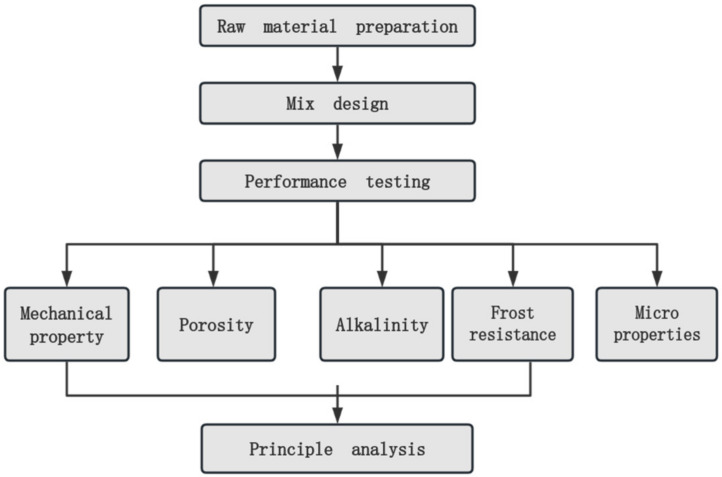
Flow process chart.

In this experiment, conch-brand (Xi’an, Shaanxi province, China) P.O 42.5 cement was used, and its physical properties are shown in Table 1. In order to replace part of the cement and reduce the alkalinity of the green-growing concrete, the solid waste is reused. The waste ACBs that were gathered from the site were crushed using an impact crusher, pulverized using a ball mill, and sieved through a standard 0.15 mm sieve to a fine powder of 0–0.15 mm. The coffee grounds collected from ordinary coffee shops were dried at 100 °C for 24 h to obtain dry coffee grounds. The SCG ash was calcined at 800 °C in a high-temperature furnace instead of partial cement. The prepared ACB powder and SCG ash samples and SEM images are shown in Figure 1. The Fourier transform infrared spectroscopy (FTIR) test was carried out using an infrared spectrometer, and the results are shown in Figure 2. The physical properties of ACBs and SCG ash are shown in Table 2 and Table 3. The chemical composition of ACBs and SCG ash are shown in Table 4 and Table 5. Limestone gravel was used as coarse aggregate, the particle size range was 4.75~9.5 mm, the close packing density was 1432 kg/m^3^, and the crushing index was 9.5%. A polycarboxylate superplasticizer with a water reduction rate of 30% was used to improve paste fluidity. The water used in this experiment is laboratory tap water. The workflow chart of this paper is shown in Figure 3.

### 2.2. Mix Proportion and Specimen Preparation

In this experiment, the volume method [27] was used to design the green-growing concrete mix ratio. The water–binder ratio was set to 0.3, and the mix ratio was cementitious material–stone–water = 1:5:0.3. The treated ACB powder and SCG ash were used to replace 0%, 5%, 15%, and 25% and 0%, 5%, 10%, and 15% cement, respectively. The test mix ratio is shown in Table 6.

### 2.3. Formation and Maintenance

The mixing process of this test was carried out according to GB/T 50081-2019 [28]. The mixing time was 150 s, and the forced single horizontal shaft concrete mixer was used for mixing [29]. The method of semi-manual vibration and semi-mechanical vibration is adopted. The artificial vibration is carried out according to JC/T2557-2020 [30], which is loaded into a 100 × 100 × 100 mm^3^ iron mold. Each layer is inserted 15 times from the edge to the center, and then, the installed mold is placed on the concrete vibration table for mechanical vibration for 5–10 s. The prepared specimens were covered with a film and placed in a standard curing room (relative temperature of 20 ± 2 °C, humidity of 95%) for curing to a specified age after 48 h of demolding.

### 2.4. Activity Index

In order to verify the activity of aerated block powder and coffee ground ash, the activity index was measured according to JG/T486-2015 [31]. The size of the specimen was the standard mortar size, and the activity index of cement mortar was tested after curing for 7d and 28d. The calculation formula for the activity index is shown in Equation (1).

(1) $$A=\frac{R_{t}}{R_{0}}\times 100$$

where A is the activity index of composite mineral admixture, in %; R_t_ is the compressive strength of the corresponding age of the tested mortar, and the unit is MPa; R_0_ is the compressive strength of the corresponding age of the contrasting mortar, and the unit is MPa.

### 2.5. Porosity

In order to ensure that the pores of the green-growing concrete meet the growth requirements of the plant, according to CJJ/T 253-2016 [32], the specimen size was 100 × 100 × 100 mm^3^, and the calculation formula for porosity is shown in Equation (2).

(2) $$C_{viod}=\left[1-\frac{m_{2}-m_{1}}{\rho V}\right]\times 100\%$$

where C_viod_ is continuous porosity, in %; m_1_ is the mass of the specimen in water, in g; m_2_ is the mass of the specimen after 24 h in the oven, in g; ρ is the density of water, in g/cm^3^; and V is the volume of the specimen, in cm^3^.

### 2.6. Compressive Strength

In order to ensure that the green-growing concrete has sufficient compressive strength and meets the requirements of green-growing concrete specification, the compressive strength test was carried out according to GB/T 50081. The specimen size was 100 × 100 × 100 mm^3^. The compressive strength of concrete cured for 7d and 28d was tested. The loading rate was 0.4 MPa/s until failure.

### 2.7. Alkalinity

In order to ensure that the alkalinity in the pores of the green-growing concrete met the needs of plant growth, the alkalinity was tested. After the specimen reached the corresponding age, 3 kg of water with a volume of 10 L was added to the bucket and placed in the bucket. The water did pass 2–3 cm on the surface of the specimen. A ZD-2 precision pH meter was used to measure the pH of the soaked water after it has been steeped for 24 h. The above steps were repeated until the pH value of the soaking water was stable, whereupon the measurement was stopped. The average value of three measurements was taken as the final test result.

### 2.8. Frost Resistance

**Figure 4:**
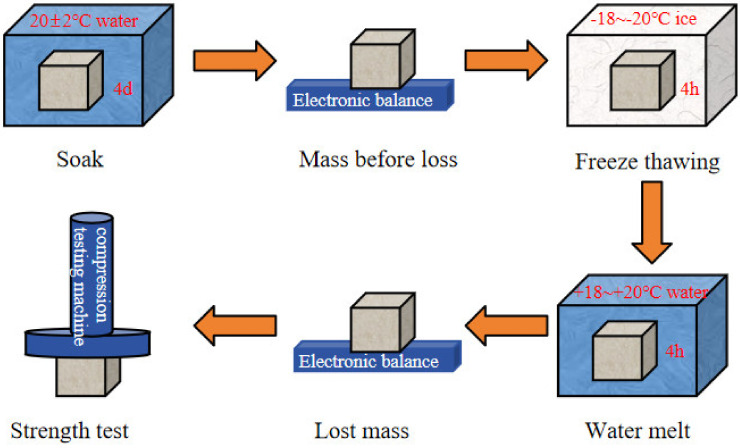
Flow chart of frost resistance of regenerated powder in green−growing concrete.

Green-growing concrete is often used for riverbank slope protection, which is greatly affected by external temperature. Therefore, it is necessary to study its frost resistance. In this paper, the slow-freezing method was used [33]. According to GB/T 50082-2009 [34], the specimen size was 100 × 100 × 100 mm^3^. Among them, the strength loss rate is as stated in Equation (3), and the mass loss rate is as stated in Equation (4). The flow chart of frost resistance is shown in Figure 4.

(3) $${∆f}_{c}=\frac{f_{c0}-f_{cn}}{f_{c0}}\times 100$$

where Δf_c_ is the loss rate of concrete compressive strength after N freeze–thaw cycles, in %; f_c0_ is the average compressive strength of the three standard curing concrete specimens for comparison, in MPa; and f_cn_ is the average compressive strength of three concrete specimens after N freeze–thaw cycles, in MPa.

(4) $${∆W}_{n}=\frac{W_{0}-W_{n}}{W_{0}}\times 100$$

where ΔW_n_ is the mass loss rate of concrete after N freeze–thaw cycles, in %; W_0_ is the mass of concrete specimen before the freeze–thaw cycle test, in g; W_n_ is the mass of concrete specimen after N freeze–thaw cycles, in g.

### 2.9. SEM Analysis

In order to reflect the influence of ACB powder and SCG ash on the properties of green-growing concrete from the microscopic point of view, the concrete containing ACB powder and SCG ash was analyzed via scanning electron microscopy (SEM). SEM was used to examine the microstructure of recycled fine powder to better understand its performance and how it influences cement hydration products. Each group of concrete samples was cut into small pieces, and then the SEM samples were dried in an oven at 103 ± 2 °C. Before being scanned, the specimens were examined under an optical microscope to determine the location of recycled fine powder particles in the concrete. According to the morphological differences of ACB powder, SCG ash, and cement hydration products, different particles’ positions were determined. Next, SEM equipment examined the shape of the regenerated micro-powder and its interaction with the cement hydration products.

## 3. Test Results and Analysis

### 3.1. FTIR Test

The raw material FTIR test of ACB powder and SCG ash is shown in Figure 4. The FTIR results further showed that SCGH ash contained abundant functional groups, and the broad peak around 3450 cm^−1^ was the absorption peak of -OH stretching vibration, which could be attributed to phenols or carboxyl group substances in SCG ash. The more substantial peak at 2820 cm^−1^ was the stretching vibration peak of -CH_3_ in the cellulose structure. The absorption peak at 1593 cm^−1^ comes from the C=C stretching vibration peak of sugar or cellulose. At 1110 cm^−1^ was a peak caused by C-O single bonds in cellulose and hemicellulose. At 774 cm^−1^ was a weak absorption peak corresponding to the C-H bending vibration peak, indicating that coffee grounds might contain some aromatic ring substances [35].

In the FTIR of ACB powder, the peak near 3580 cm^−1^ belonged to the -OH stretching vibration peak, and an obvious absorption band could be observed on this peak. At 3300–3200 cm^−1^, there was a broad stretching vibration peak of -OH. At 1427 cm^−1^ and 877 cm^−1^, there were the stretching vibration peaks of -CO_3_^2−^, which indicated that multiple calcium carbonate polycrystalline materials might be formed. At 1160 cm^−1^ and 970 cm^−1^, were the main structures of Si−O bonds in C−S−H gels. With the increase in carbonization degree, the vibratory position of Si−O bonds gradually moved to a higher wave number, indicating that the silicates of C−S−H extend after carbonization [36].

### 3.2. Activity Index

The activity index [37] of ACB powder and SCG ash cement mortar is shown in Table 7. From the table, it is shown that the strength of ACB powder 7d and 28d gradually increases, at 7d rises fastest, and its activity index reaches 85.3%. This showed that cement mortar containing ACB powder rapidly increases strength at 30% ACB powder content, indicating that ACB powder has excellent potential for utilization in cement mortar [12]. Although the activity index of SCG ash at 7d and 28d increased, the rising trend was slow, and the strength was not high. Therefore, the activity index of SCG ash (7d) was much lower than that of ACB powder. The activity index of SCG ash (7d) only reached 39.7%. This was because SCG ash absorbs a large amount of water during the hydration process, resulting in insufficient water being involved in the hydration reaction, so its strength was low.

### 3.3. Porosity

By varying the amount of ACB powder and SCG ash with a fixed water–binder ratio of 0.3, the impact of these materials on the green-growing concrete’s porosity was investigated [38,39]. The specific test results are shown.

**Figure 5:**
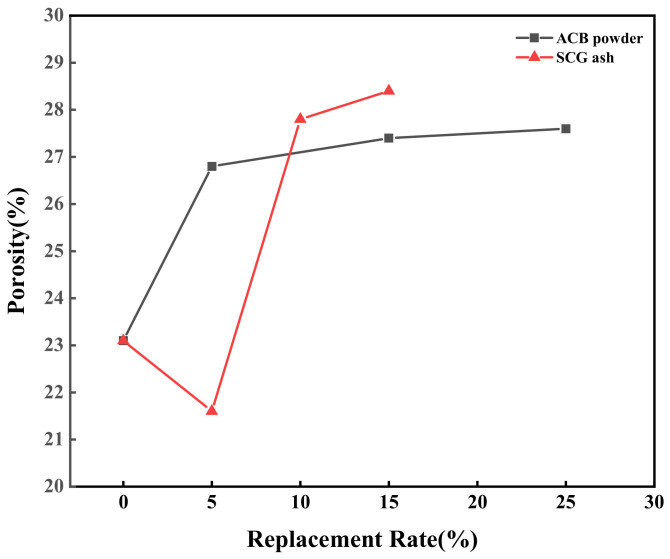
The porosity of recycled fine powder concrete.

It can be seen from Figure 5 that the porosity of the control group was 23.1%. The concrete of each group with ACB recycled fine powder was higher than that of the control group. In addition, with the increase in ACB powder content, the porosity of concrete containing ACB powder increases, and the growth rate of concrete samples containing 5% ACB powder was the largest, at 16.0%. This indicated that ACB particles have micropores [40], consistent with the results observed in the SEM image in Figure 1. With the increase in SCG ash content, the porosity of concrete containing SCG ash decreases first and then increases. When the content of SCG ash was 5%, the porosity of concrete containing SCG ash was 21.6%, which was 6.5% less than that of the control group. When the content of SCG ash was 10%, the porosity of concrete containing SCG ash was 27.8%, which was 20.3% greater than that of the control group. This demonstrated that while a high SCG ash concentration would not support the development of concrete porosity, a small quantity of SCG ash has a micro-filling impact that could dramatically lower the porosity of cement-based composites [18]. The porosity of the ACB-SCG powder regenerated powder doped with 5% was 24.9%, which indicated that ACB powder and SCG ash would fill a part of the pores after co-doping, which could balance the pores of the two raw materialsare single-doped.

### 3.4. Compressive Strength

**Figure 6:**
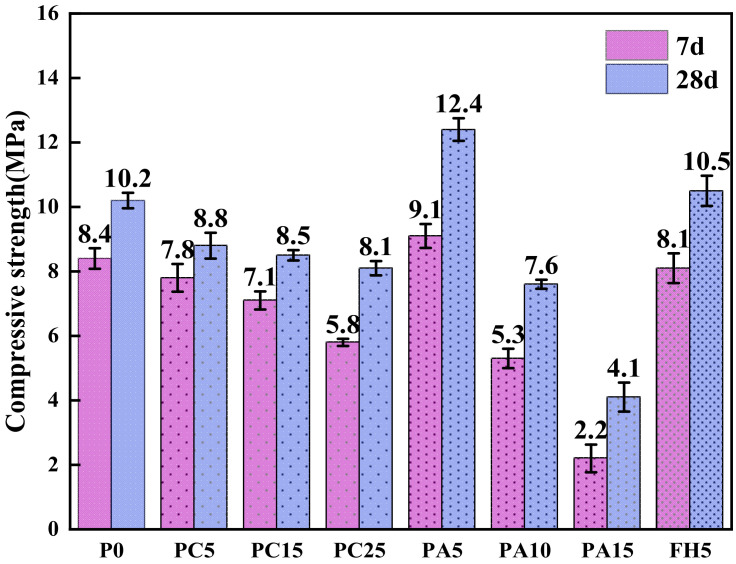
The compressive strength of concrete with recycled fine powder.

Three control group specimens and three other group specimens were prepared for the compressive strength test. The average strength of each group, together with the error bar representing the standard deviation, is shown in Figure 6. When the substitution rate of ACB powder in the green-growing concrete increased from 0% to 25%, the 7d compressive strength decreased from 8.4 MPa to 5.8 MPa, by 31.0%, and the 28d compressive strength decreased from 10.2 MPa to 8.1 MPa, by 20.6%. The reason for this was that, with an increase in substitution rate, the secondary hydration reaction of ACB powder dramatically reduced the formation of Ca(OH)_2_ crystal and C-S-H gel, which caused a decrease in the compressive strength of the green-growing concrete containing ACB powder [12]. Because the green-growing concrete was a kind of porous concrete, the compressive strength of ACB powder was not as high as the results of the above review, and the optimal content was 5%. Nevertheless, the concrete meets China’s planting concrete standard, JC/T2557-2020. With the increase in SCG ash substitution rate, the compressive strength of the green-growing concrete increases first and then decreases. When the substitution rate increased from 0% to 5%, the 7d compressive strength increased from 8.4 MPa to 9.1 MPa, an increase of 8.3%, and the 28d compressive strength increased from 10.2 MPa to 12.4 MPa, an increase of 21.6%. This was because the coffee grounds form a crystal-like substance after combustion, and a small amount of SCG ash could fill the interface between the cement slurry and the aggregate, increasing the compressive strength [17]. This was consistent with the results of Choi [18], and the best dosage was 5%. Due to the increased replacement rate, more SCG ash pores could absorb free water from concrete. This prevented hydration without considering the pozzolanic activity of SCG ash, significantly decreasing the concrete’s compressive strength.

### 3.5. Alkalinity

**Figure 7:**
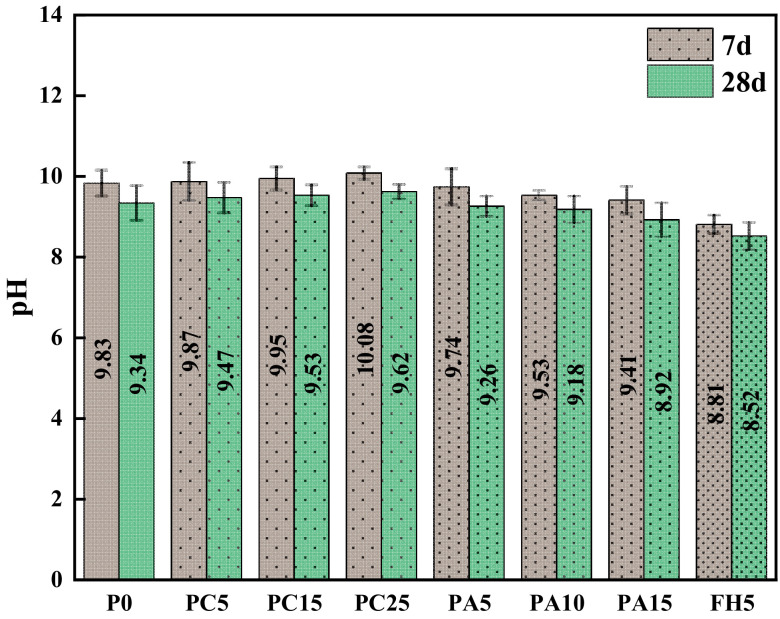
Alkalinity of recycled fine powder concrete.

Figure 7 shows the pH values of the green-growing concrete 7d and 28d under different ACB powder and SCG ash contents. Each value was the average value of the three samples and the error bar in the group. With the increase in ACB powder content, the alkalinity of concrete containing ACB powder increases, but it was roughly between 9 and 10. This showed that adding ACB powder could reduce the alkalinity of concrete. This was because, as the content of ACB powder increased, less cement was used, which lowered the alkalinity of the concrete. However, since the aerated block was also made up of cement, lime, gypsum, and other alkalinity-containing cementitious materials, the alkalinity of the concrete that contains ACB powder rises [22]. With the increase in SCG ash content, the alkalinity of SCG ash-containing concrete decreases, to roughly between 9 and 10. This showed that adding SCG ash could effectively reduce the alkalinity of concrete. This was because, with the increase in SCG ash content, the amount of cement decreases, resulting in a decrease in concrete alkalinity. SCG ash is a kind of biochar that does not have alkalinity. Hence, the alkalinity of the green-growing concrete decreases [23]. The green-growing concrete alkalinity of ACB-SCG powder was lower, only 8.52 at 28d, saving cement and effectively reducing the alkalinity.

### 3.6. Frost Resistance

**Figure 8:**
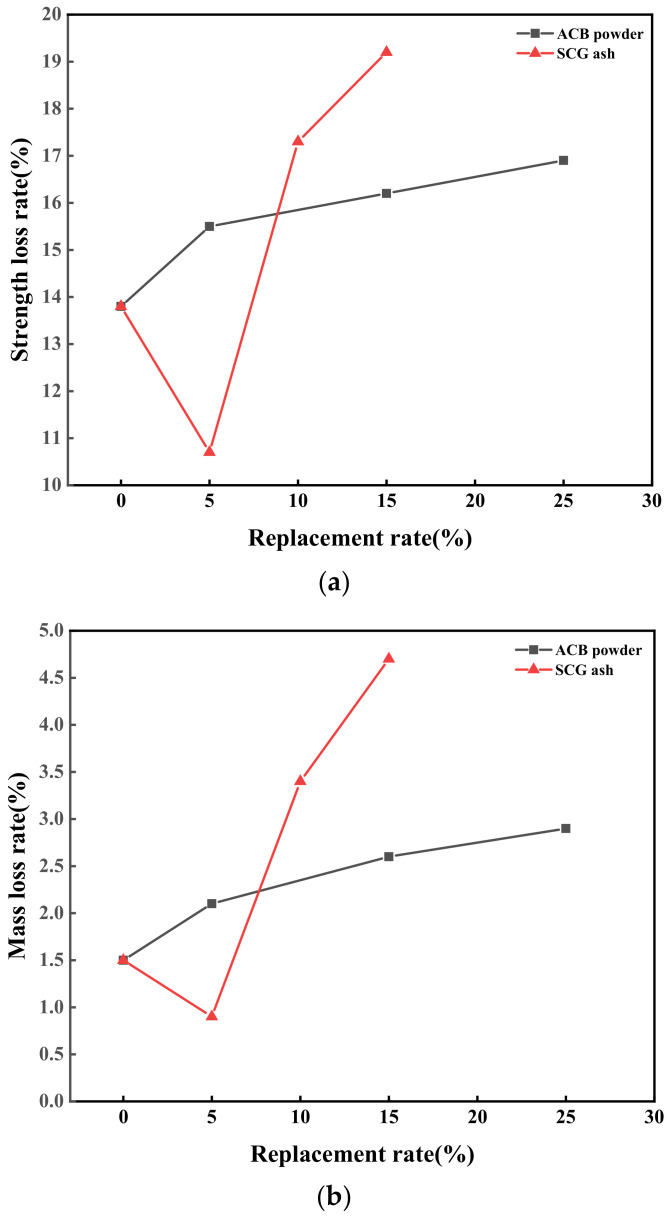
Frost resistance of recycled fine powder concrete. (**a**) Strength loss rate. (**b**) Mass loss rate.

The slow-freezing method was used to examine the impact of various recycled powder replacement rates on the green-growing concrete’s mass and compressive strength loss rates under freeze–thaw conditions. Figure 8 displays each variable’s mass and compressive strength loss rates.

It can be seen from Figure 8a that with the increase in ACB powder content, the strength loss rate of concrete containing ACB powder within 25 freeze–thaw cycles gradually increases. Among them, the strength loss rate of the control group was 13.8%. The strength loss rate of concrete with 25% ACB powder was 16.9%, 22.5% higher than that of the control group. In addition, it could be clearly seen that the strength loss rate of concrete samples containing ACB powder was more significant than that of concrete in the control group. The micropores in the ACB powder made it simple for pore water to be absorbed, which caused internal water loss and a greater rate of strength loss at a higher replacement rate [36]. The strength loss rate of concrete containing 5% SCG ash powder was 10.7%, 22.5% less than that of the control group. The strength loss rate of concrete containing 10% SCG ash powder was 17.3%, 25.4% higher than that of the control group. This showed that a small amount of SCG ash has a micro-filling effect, which could reduce the porosity. More SCG ash absorbs ample internal water, increasing the strength loss rate. The strength loss rate of 5% ACB-SCG powder was 14.6% because a small amount of SCG ash could fill the pores inside the concrete and slightly increase the strength. The planting concrete (JCT 2557-2020) specification requires that the strength loss rate be less than or equal to 20% and that the test meet its requirements.

Figure 8b shows the mass loss rate of ACB powder and SCG ash concrete specimens. Within 25 freeze–thaw cycles, the mass loss rate of concrete containing ACB powder gradually increases. Among them, the mass loss rate of the control group was 1.5%, and the mass loss rate of concrete containing 25% ACB powder was 2.9%, which was 1.4% higher than that of the control group. This was due to the porosity of the green-growing concrete itself, and ACB powder absorbs a lot of water during the hydration process to make the pores more prominent. Due to freeze–thaw damage, the original closed pores and semi-connected pores in the concrete become connected pores, so the green-growing concrete pores could not hold water, decreasing the quality of the test block [41]. The mass loss rate of concrete containing 5% SCG ash was 0.9%, 0.6% lower than that of the control group. The mass loss rate of concrete containing 10% SCG ash was 3.4%, 1.9% higher than that of the control group. This indicated that a small amount of SCG ash filled the pores inside the concrete, increasing its quality. Still, too much SCG ash absorbs a large amount of water during the hydration process to make the pores larger, increasing the internal pores of the concrete and substantially reducing the quality of the concrete [42]. The mass loss rate of ACB-SCG powder with a 5% ACB powder content was 1.8%, which was lower than that of ACB powder with a 5% ACB powder content. According to the standard for planting concrete (JCT 2557-2020), the mass loss rate is less than or equal to 5%, and the test meets its requirements.

### 3.7. Microstructure Analysis

**Figure 9:**
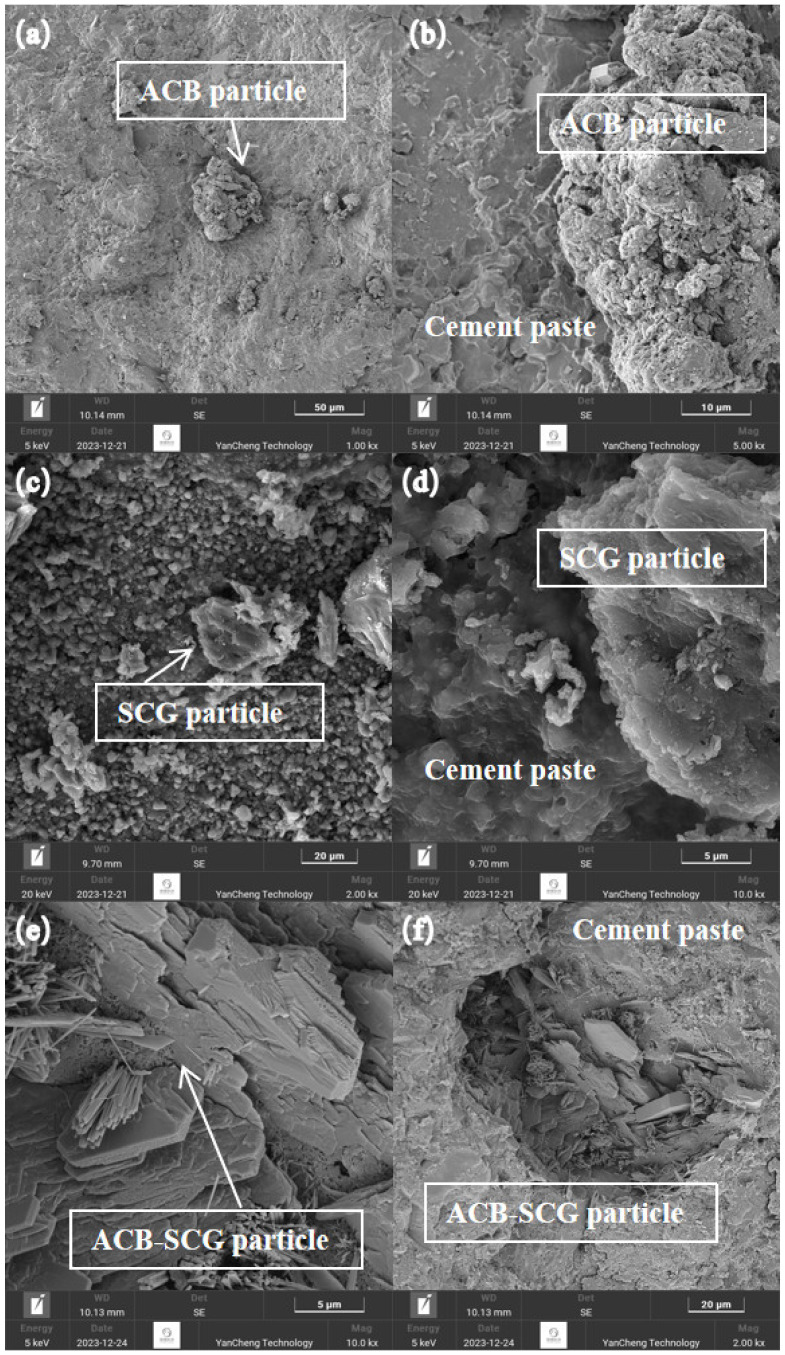
Micromorphology of concrete containing recycled fine powder: (**a**) optical microscope photo of ACB particle; (**b**) interface transition zone between ACB particle and cement paste; (**c**) optical microscope photo of SCG ash; (**d**) interface transition zone between SCG ash and cement paste; (**e**) optical microscope photo of ACB-SCG powder; and (**f**) interface transition zone between ACB-SCG powder and cement paste.

Figure 9 shows the SEM diagram of concrete containing ACB powder and SCG ash after curing for 28d. Figure 9a shows the ACB particles. The cement slurry was enlarged to obtain Figure 9b. Figure 9b shows the SEM image of the interface transition zone between ACB particles and cement slurry. It can be seen from Figure 9b that many ACB particles were distributed in the cement slurry. The ACB particles contained micropores and were closely connected to the cement slurry interface without an apparent loose texture. This might be due to the decreasing effective water–cement ratio on the surface of ACB particles after the micropores absorb water. Therefore, the hydration products of cement were denser [40]. SCG ash particles are shown in Figure 9c, and cement slurry could be amplified to produce Figure 9d. Figure 9d shows the SEM diagram of the interfacial transition zone between SCG ash particles and cement slurry. It can be seen from Figure 9d that many SCG ash particles were distributed in the cement paste due to the micro-hydration reaction between SCG ash particles and cement, the interface between the aggregate and cement paste of SCG ash concrete test block is relatively dense [18]. Figure 9e shows the SEM image after compounding 5% ACBs and SCGs. The diagram shows that there were many flaky and microporous substances in the cement slurry, which was the ACB-SCG powder. There was a small amount of needle-like fiber material, which was the hydration product of cement ettringite (Ca_6_Al_2_(SO_4_)_3_(OH)_12_·26H_2_O). Figure 9f shows the SEM diagram of the interface transition zone between ACB-SCG powder and cement slurry. It can be seen from the diagram that the interface between the ACB-SCG powder and the cement slurry was dense.

## 4. Conclusions

The following conclusions can be drawn in experimental settings based on the examination of the characteristics of ACB powder, SCG ash regenerated powder, and ACB-SCG powder and how they affected the performance of green-growing concrete:

(1) It can be observed from the SEM images of ACB powder and SCG ash that the surface of ACB particles contains many micropores, while the surface of SCG ash particles is serrated and wrinkled. The surface of ACB-SCG particles has microporous, blocky, and needle-like fibers.
(2) The addition of ACB powder significantly increases the porosity of concrete, up to 27.6%; meanwhile, the porosity of concrete containing SCG ash decreases first and then increases, up to 28.4%. ACB-SCG powder can balance the porosity of both single-doped concretes. For all concrete containing recycled fine powder, ACB powder and SCG ash replacing 5% cement can improve the strength of cement-based composites.
(3) The alkalinity of concrete containing ACB powder in 7d and 28d gradually increases, but the basicity of 28d is slightly lower than that of 7d, and the lowest alkalinity of 28d is 9.47; the alkalinity of concrete containing SCG ash in 7d and 28d gradually decreases, and the alkalinity of 28d s slightly lower than that of 7d, and the lowest alkalinity of 28d is 8.92. ACB-SCG powder has a lower alkalinity than ACB powder but a slightly higher alkalinity than SCG ash.
(4) The influence of ACB powder and SCG ash on anti-freezing performance was studied using the slow freezing method. The freeze-resistance of concrete containing ACB powder gradually decreases, the freeze-resistance of concrete containing SCG ash increases at first and then decreases significantly, and the best frost resistance of concrete containing SCG ash is 5% SCG ash. Meanwhile, the freeze-resistance of ACB-SCG powder neutralizes the performance of ACB powder and SCG ash, respectively.
(5) In the future, it will be possible to chemically treat coffee grounds and adopt different curing methods to greatly increase their compressive strength, so as to explore the possibility of its application on architectural landscape walls.

## Figures and Tables

**Table 1:** Main performance parameters of cement.

Specific Surface Area m^2^/kg	Density g/cm^3^	Setting Time/min		Flexural Strength/MPa		Compressive Strength/MPa	
		Initial Set	Final Set	3d	28d	3d	28d
458	2.76	98	142	3.54	7.57	21.98	44.83

**Table 2:** Physical properties of ACBs.

Bulk Density g/cm^3^	Apparent Density g/cm^3^	Porosity/%
0.790	2.584	68.5

**Table 3:** Physical properties of SCG ash.

Specific Gravity	Water Absorption/%
0.75	15

**Table 4:** Chemical constituents of ACBs.

Oxides	CaO	SiO_2_	Al_2_O_3_	Fe_2_O_3_	MgO	SO_3_	Loss on Ignition/%
Content/%	21.7	54.8	5.8	2.6	1.2	4.5	9.4

**Table 5:** Element analysis of coffee residue and ash.

Items	Element				
	Carbon (C)	Nitrogen (N)	Hydrogen (H)	Oxygen (O)	Sulfur (S)
SCG (%)	53.14	4.23	1.09	41.51	0.03
SCG ash (%)	57.69	2.49	14.98	24.83	0.01

**Table 6:** The w/c ratio is 0.3 for green-growing concrete mix design.

Numbering	Cement/kg	ACB Powder/g	SCG Ash/g	Gravel/kg	Water/kg	Water Reducer/g
P0	6	0	0	30	1.8	12
PC5	5.7	300	0	30	1.8	12
PC15	5.1	900	0	30	1.8	42
PC25	4.5	1500	0	30	1.8	72
PA5	5.7	0	300	30	1.8	24
PA10	5.4	0	600	30	1.8	60
PA15	5.1	0	900	30	1.8	210
FH5	5.4	300	300	30	1.8	12

**Table 7:** Activity index of ACB powder and SCG ash cement mortar.

Number	Strength/MPa		Activity Index/%	
	7d	28d	7d	28d
Control	36	43	-	-
ACB powder	30.7	38.5	85.3	89.5
SCG ash	14.3	20.6	39.7	47.9

## Data Availability

Data are contained within the article.
